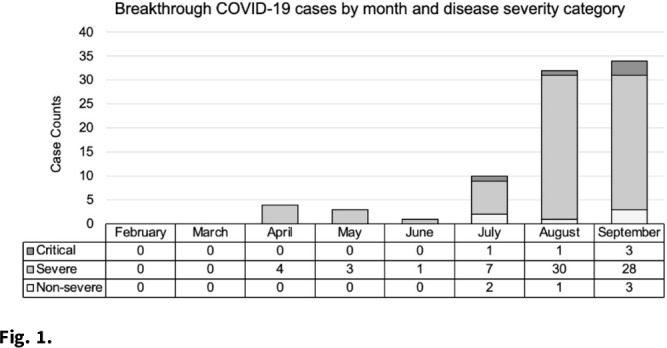# SARS-CoV-2 breakthrough infections among hospitalized patients in southeastern Michigan

**DOI:** 10.1017/ash.2022.133

**Published:** 2022-05-16

**Authors:** Sydney Fine, Kellee Necaise, Alexandra Hayward, Anurag Malani

## Abstract

**Background:** As of January 2022, more than 57 million cases of COVID-19 have been reported in the United States. Three primary COVID-19 vaccines are widely available: Pfizer (BNT162b2), Moderna (mRNA-1273), and Johnson & Johnson’s-Janssen (JNJ-78436735). The vaccines are effective but do not prevent all infections. We investigated trends in type of vaccine receipt, demographic characteristics, and disease outcomes in COVID-19 breakthrough infections among hospitalized patients. **Methods:** A breakthrough case is defined as the detection of SARS-CoV-2 ≥14 days after completion of all doses of an FDA-authorized COVID-19 vaccine. An electronic medical record report in EPIC EHR software identified 85 fully vaccinated patients with a documented positive SARS-CoV-2 result between February and September 2021 at 2 hospitals in southeastern Michigan. Demographic information and hospitalization characteristics, including length of stay and oxygen requirements, were collected from the report. Patients were classified into disease severity categories: nonsevere, severe, or critical. A case was considered severe if the patient’s oxygen saturation level (SpO_2_) was ≤94% on room air or if the patient required supplemental oxygen. Illness was considered critical if the patient developed respiratory failure, including mechanical ventilation or extracorporeal membrane oxygenation. All other cases were classified as nonsevere. Cycle threshold (Ct) values, the number of PCR cycles required to reach a threshold of SARS-CoV-2 genomic material, were collected from the hospital microbiology lab. **Results:** We identified 85 breakthrough infections (Fig. 1). The average patient age was 69.9±15.7 years, and 44 (51.8%) were female. Severe disease was most common (n = 73, 85.9%) followed by nonsevere disease (n = 7, 8.24%), and 9 patients (10.6%) in this cohort died. Most patients received either the Moderna (n = 35, 41.2%) or Pfizer (n = 38, 44.7%) vaccines. Pfizer vaccine receipt was most common among patients with severe illness (n = 33 of 73, 45.2%), and Moderna vaccine receipt was most common among patients with critical illness (n = 4 of 5, 80.0%). Average time from last vaccination to positive test was longest among Moderna vaccine recipients (181.9±43.1 days) and shortest among J&J vaccine recipients (91.0±61.1 days). The average Ct value was 23.8±7.5 and ranged from 13.0 to 41.3. There were no appreciable differences in the average Ct value by vaccine manufacturer. **Conclusions:** Breakthrough infections among hospitalized patients were uncommon, but incidence increased with time after vaccine receipt in all vaccines. Further study is needed to examine differences and severity in breakthrough infections by vaccine type and in individuals who completed booster vaccines.

**Funding:** None

**Disclosures:** None

**Figure:**